# Etiological characteristics and risk factors for severe disease in bocavirus-associated community-acquired pneumonia in children: a multicenter retrospective study

**DOI:** 10.1186/s12879-026-12543-z

**Published:** 2026-01-27

**Authors:** Yuqi Wang, Zhenmei Wei, Chan Su, Junmei Fang, Zhong Hu, Yingshuo Wang, Zhimin Chen

**Affiliations:** 1 Department of Pulmonology, Children’s Hospital, Zhejiang University School of Medicine, National Clinical Research Center for Children and Adolescents’ Health and Diseases, No. 3333, Binsheng Road, Binjiang District, Hangzhou City, Zhejiang Province 310052 PR China; 2 Department of Pediatrics, Jinhua Maternal and Child Health Hospital, Jinhua, PR China; 3 Department of Pediatrics, Dongyang Maternal and Child Health Hospital, Dongyang, PR China; 4 Department of Pediatrics, Yongkang Maternal and Child Health Hospital, Yongkang, PR China

**Keywords:** Bocavirus, tNGS, Community-acquired pneumonia, Children, Severe pneumonia

## Abstract

**Objective:**

This is a multicenter retrospective study aimed to evaluate the concordance between nucleic acid testing and targeted next-generation sequencing (tNGS) for etiologic detection, and to identify potential risk factors for severe community-acquired pneumonia (SCAP), thereby providing evidence for precision clinical management.

**Methods:**

We retrospectively analyzed 191 pediatric Community-Acquired Pneumonia(CAP) cases positive for bocavirus by tNGS from April 2023 to June 2025. Etiologic concordance and coinfection patterns were evaluated. Univariate and multivariate logistic regression identified independent risk factors for SCAP.

**Results:**

191 children were included. Significant heterogeneity was observed among the four centers in age distribution, SCAP incidence, imaging findings, and treatment patterns (*P* < 0.05). Children’s Hospital, Zhejiang University School of Medicine (ZCH) reported the highest SCAP incidence (50.0%), with markedly higher rates of pulmonary consolidation (66.7%), atelectasis (100.0%), and pleural effusion (16.7%) compared with other centers, whereas DY had the lowest SCAP incidence (11.0%). All cases were bocavirus-positive by tNGS, while the positivity rate by nucleic acid testing was 51.3%. tNGS identified 44 cases of mixed detection (23.0%), including 35 bacterial (18.3%) and 7 viral co-detections (3.7%), whereas nucleic acid testing identified only 9 mixed detections (4.7%). tNGS demonstrated a clear advantage in detecting mixed and bacterial detections (*P* < 0.001). Multivariate logistic regression revealed that age < 3 years, plastic bronchitis, and increased prealbumin were independent risk factors for SCAP (*P* < 0.05).

**Conclusion:**

Clinical profiles of bocavirus-associated CAP vary significantly across centers. tNGS offers a broader range of pathogen co-detection compared with conventional nucleic acid testing. Early recognition of high-risk children—particularly those < 3 years with potential plastic bronchitis and viral co-detection—is essential for timely SCAP management.

**Clinical trial registration:**

This multicenter retrospective study was registered with the Chinese Clinical Trial Registry (ChiCTR), a primary registry within the WHO Registry Network. The registration was completed on January 19, 2025 (Registration number: ChiCTR2400080059), with a predefined target of enrolling at least 150 cases.

**Supplementary Information:**

The online version contains supplementary material available at 10.1186/s12879-026-12543-z.

## Introduction

Community-acquired pneumonia (CAP) is a major cause of morbidity and mortality in children under five years worldwide, with severe CAP (SCAP) accounting for 10–20% of cases and contributing substantially to poor outcomes [1, 2]. Human bocavirus (HBoV), identified in 2005, has since emerged as a significant respiratory pathogen in children. Studies report detection rates up to 7% in acute respiratory infections, and growing evidence links HBoV to pneumonia, plastic bronchitis, and other severe lower respiratory tract complications [3, 4]. Although most children present with fever, cough, and wheezing, some progress rapidly to respiratory failure or extensive radiologic involvement, while the mechanisms underlying severe disease remain unclear [5].

Accurate pathogen identification is essential for targeted management of CAP. Conventional nucleic acid testing (e.g., PCR) is widely used due to its accessibility and rapid turnaround, but its reliance on predefined targets limits the detection of mixed or uncommon pathogens [5]. Targeted next-generation sequencing (tNGS) overcomes these constraints by enabling simultaneous, high-sensitivity detection of a broad range of bacteria and viruses [6]. However, its higher cost, operational complexity, and interpretative requirements restrict routine use, and its diagnostic consistency with traditional testing in HBoV-associated CAP has not been well established [7].Nevertheless, studies have demonstrated the value of tNGS in characterizing respiratory pathogens in children with acute respiratory infections [8].

To date, multicenter evidence on the clinical characteristics of HBoV-associated CAP, the comparative value of diagnostic methods, and the determinants of SCAP in children remains limited. This study analyzed pediatric cases with tNGS-confirmed HBoV infection from four centers. We aimed to characterize inter-center clinical heterogeneity, compare tNGS with nucleic acid testing for etiologic detection, and identify independent risk factors for SCAP, providing evidence to improve the clinical management of HBoV-associated CAP.

## Methods

### Study participants

This retrospective study included children diagnosed with CAP who tested positive for human bocavirus by tNGS at four medical centers in Zhejiang Province, China: Zhejiang University School of Medicine Children’s Hospital (ZCH), Yongkang Women and Children’s Hospital (YK), Jinhua Women and Children’s Hospital (JH), and Dongyang Women and Children’s Hospital (DY), between April 2023 and June 2025. Characteristics of the participating medical centers are detailed in Supplementary Table S1. Our study was approved by the institutional ethics committee (2023-IRB-0042-P-02). This multicenter retrospective study was registered with the Chinese Clinical Trial Registry (ChiCTR), a primary registry within the WHO Registry Network. The registration was completed on January 19, 2025 (Registration number: ChiCTR2400080059).

The Inclusion criteria is following: (1) Age 29 days to 16 years; (2) Diagnosis of CAP based on the Guidelines for the management of community-acquired pneumonia in children (2024 revision) [9], where CAP is defined as pulmonary infection acquired outside the hospital and meeting at least 2 of the following criteria: ① Newly occurring or exacerbation of clinical symptoms including cough, fever, or dyspnea; ② rales or wheezing on lung auscultation; ③ Radiological evidence of pulmonary inflammatory infiltration; (3) Respiratory specimens (e.g., nasopharyngeal swabs) collected after admission and subjected to both tNGS and nucleic acid testing; (4) Positive HBoV result by tNGS; (5) Complete clinical data.

Patients meeting any of the following criteria were diagnosed with SCAP [8]: respiratory failure, multilobar lung involvement, pleural effusion, altered mental status, or septic shock.

### Laboratory methods and inter-center standardization

A standardized laboratory protocol was implemented across all four centers, with ZCH providing unified guidelines to collaborative hospitals (JH, YK, DY). The key aspects of standardization are detailed below.

For specimen collection and handling, a uniform operating procedure for nasopharyngeal swab collection was followed. Samples were collected using identical synthetic fiber swabs and universal transport medium, stored at 4 °C, and processed within 2 h of collection at each center’s laboratory.

For Viral and Atypical Pathogen Detection (Nucleic Acid Testing), all centers systematically used the identical commercial multiplex real-time PCR (RT-PCR) assay for the initial screening. This assay simultaneously detects a panel of common respiratory viruses (including human bocavirus, respiratory syncytial virus, rhinovirus, etc.) and atypical pathogens (e.g., Mycoplasma pneumoniae). The same PCR platforms and reagent batches were employed to minimize inter-laboratory variability. For Broad-pathogen Detection (tNGS), all centers adopted the same targeted next-generation sequencing (tNGS) kit and sequencing platform (e.g., Illumina) for definitive and broad-spectrum pathogen identification. From nucleic acid extraction to bioinformatic analysis, a consistent pipeline was followed as per the manufacturer’s protocol and the study’s unified guideline.

All enrolled children with CAP underwent both the standardized multiplex RT-PCR and tNGS testing as part of the agreed-upon diagnostic workup, ensuring a systematic and comparable detection effort across all sites. All laboratories adhered to stringent internal quality control measures. Each run of RT-PCR and tNGS included external positive and negative controls. Furthermore, the four laboratories regularly participate in and have passed the same external quality assessment programs for molecular diagnostics, ensuring a high level of consistency in assay performance and interpretation across centers.

### Data collection

Clinical data were extracted from electronic medical records, including demographics (age, sex, height, weight, time of onset, study center, and medical history), clinical symptoms (fever, cough, wheezing, dyspnea, and lung auscultation finding), laboratory tests (white blood cell count, neutrophil and lymphocyte percentages, eosinophils, platelets, hemoglobin, C-reactive protein, procalcitonin, prealbumin, globulin, total bilirubin, Alanine Aminotransferase(ALT), Aspartate Aminotransferase(AST), creatinine, urea nitrogen, Lactate Dehydrogenase(LDH), creatine kinase, and Immunoglobulin E (IgE)), imaging (chest radiography, chest computed Tomography (CT), and bronchoscopy), etiologic findings (results from tNGS and nucleic acid testing), and interventions (oxygen therapy or ventilatory support, antimicrobial treatment, and corticosteroid use).

### Statistical analysis

Continuous variables with normal distribution were expressed as mean ± standard deviation (SD) and compared using the *t*-test (two groups) or One-way ANOVA (four groups); non-normally distributed data were described as median (interquartile range, IQR) and compared using the Mann–Whitney U test (two groups) or Kruskal-Wallis H test (four groups). Categorical variables were presented as frequency (n) (percentage, %) and compared using the χ² test or Fisher’s exact test.

Potential risk factors for SCAP were first screened by univariate logistic regression. Variables with *P* < 0.10 were included in a multivariable logistic regression model adjusting for sex, body mass index, allergy history, white blood cell count, and other confounders. Odds ratios (OR) and 95% confidence intervals (CI) were reported. A two-sided *P* < 0.05 indicated statistical significance. All analyses were performed by R version 4.3.2.

## Results

### Clinical characteristics

A total of 191 children were included, comprising 125 males (65.4%) and 66 females (34.6%), with a mean age of 25.63 months. The distribution of cases among the four centers was as follows: DY, 73 (38.2%); JH, 35 (18.3%); YK, 65 (34.0%); and ZCH, 18 (9.4%). Significant inter-center differences were observed in age distribution, clinical presentation, incidence of severe pneumonia, pathogen detection results, and treatment strategies. The baseline clinical characteristics of children from the four centers are summarized in Table 1.

The majority of patients were aged 1–3 years (58.6%), followed by those aged 3–6 years (26.2%). Children younger than 1 year accounted for 12.0%, while only 3.1% were older than 6 years. The mean age differed significantly among the centers, with the oldest patients from JH (34.39 months) and the youngest from ZCH (22.13 months) (*P* = 0.038). Seasonal variation was evident, with the highest incidence in summer (48.2%), followed by autumn (25.7%), and a statistically significant difference across seasons (*P* = 0.014).

The most common symptoms were cough (98.4%) and fever (83.2%; mean peak 38.53 °C), with no significant inter-center differences. Wheezing occurred in 31.4% and dyspnea in 17.3% of patients, most frequently at ZCH (33.3%) and least at DY (9.6%). Mean White Blood Cell Count (WBC) was 9.63 × 10⁹/L, highest at YK (13.19 × 10⁹/L) and lowest at DY (6.26 × 10⁹/L) (*P* < 0.001). C-Reactive Protein(CRP) and Procalcitonin(PCT) were elevated at JH/ZCH (CRP: 10.50/9.64 mg/L, *P* = 0.022; PCT: 0.95 ng/mL at ZCH, *P* = 0.034). ZCH patients also had higher IgE (211.71 IU/mL) and higher rates of eosinophilia (27.8%) and allergy history (22.2%) compared with other centers (*P* < 0.001).

Among the 191 patients, 42 (22.0%) developed SCAP. SCAP incidence was highest at ZCH (50.0%) and JH (37.1%), and lower at YK (18.5%) and DY (11.0%) (*P* < 0.001). Radiologically, the overall rates of pulmonary consolidation and atelectasis were 16.8% and 18.3%, respectively, with ZCH showing markedly higher rates of consolidation (66.7%) and atelectasis (100.0%) (*P* < 0.001). Pleural effusion occurred in 2.1% of cases, detected only at ZCH (16.7%) and YK (1.5%) (*P* < 0.001). Plastic bronchitis observed via bronchoscopy was most frequent at ZCH (22.2%), and lower at DY (2.7%) and YK (3.1%) (*P* = 0.007).

Regarding etiology, 79.1% of patients had HBoV mono-detection, 18.3% had bacterial co-detection, and 3.7% had viral co-detection, with significant inter-center differences (*P* < 0.001). Bacterial co-detection was most common at JH (37.1%) and DY (23.3%), viral co-detection was highest at YK (9.2%), and no co-detections were detected at ZCH (*P* < 0.001).

Treatment mainly included cephalosporins, penicillin, azithromycin, and corticosteroids. Cephalosporins were the most commonly used antibiotics (43.5%), with similar usage across centers. Azithromycin use was highest at DY (71.2%), while penicillin was lowest (1.4%) (*P* < 0.001). Overall methylprednisolone use was 49.2%, lowest at YK (35.4%), with over half of patients at the other centers receiving treatment (*P* = 0.044). A small proportion (4.7%) received neither antibiotics nor corticosteroids, more frequently at JH (17.1%) and ZCH (11.1%) (*P* < 0.001).

**Table 1:** Clinical characteristics of included participants

Variates	Overall	DY	JH	YK	ZCH	*P*
n	191	73	35	65	18	
Age, month (mean (SD))	25.63 (20.21)	24.59 (14.77)	34.39 (26.76)	23.06 (22.42)	22.13 (10.69)	0.038
Age group (n (%))						0.524
< 1 year	23 (12.0)	7 (9.6)	4 (11.4)	11 (6.9)	1 (5.6)	
1–3 years	112 (58.6)	44 (60.3)	17 (48.6)	38 (58.5)	13 (72.2)	
3–6 years	50 (26.2)	21 (28.8)	12 (34.3)	13 (20.0)	4 (22.2)	
> 6 years	6 (3.1)	1 (1.4)	2 (5.7)	3 (4.6)	0 (0.0)	
Sex	66 (34.6)	23 (31.5)	14 (40.0)	22 (33.8)	7 (38.9)	0.82
BMI (mean (SD))	16.73 (2.50)	17.26 (2.25)	16.25 (3.59)	16.58 (2.02)	16.06 (2.21)	0.107
Morbidity season (n (%))						0.014
Spring	34 (17.8)	10 (13.7)	4 (11.4)	19 (29.2)	1 (5.6)	
Summer	92 (48.2)	33 (45.2)	23 (65.7)	27 (41.5)	9 (50.0)	
Fall	49 (25.7)	26 (35.6)	4 (11.4)	12 (18.5)	7 (38.9)	
Winter	16 (8.4)	4 (5.5)	4 (11.4)	7 (10.8)	1 (5.6)	
Severe pneumonia (%)	42 (22.0)	8 (11.0)	13 (37.1)	12 (18.5)	9 (50.0)	< 0.001
History of allergies (%)	10 (5.2)	0 (0.0)	5 (14.3)	1 (1.5)	4 (22.2)	< 0.001
Oxygen inhalation (%)	35 (18.3)	16 (21.9)	7 (20.0)	7 (10.8)	5 (27.8)	0.236
clinical picture						
Fever (%)	159 (83.2)	64 (87.7)	32 (91.4)	48 (73.8)	15 (83.3)	0.078
Average number of fever days (mean (SD))	2.49 (2.63)	2.29 (1.93)	2.40 (1.95)	2.29 (2.73)	4.22 (4.73)	0.033
Highest body temperature (mean (SD))	38.53 (1.05)	38.55 (0.88)	38.82 (1.00)	38.31 (1.19)	38.73 (1.16)	0.107
Cough (%)	188 (98.4)	72 (98.6)	35 (100.0)	63 (96.9)	18 (100.0)	0.611
Coughing with wheezing (%)	60 (31.4)	20 (27.4)	10 (28.6)	25 (38.5)	5 (27.8)	0.515
Difficulty in breathing (%)	33 (17.3)	7 (9.6)	9 (25.7)	11 (16.9)	6 (33.3)	0.046
Wheezing sound (%)	78 (40.8)	40 (54.8)	10 (28.6)	21 (32.3)	7 (38.9)	0.018
Rales (wet) (%)	86 (45.0)	20 (27.4)	10 (28.6)	50 (76.9)	6 (33.3)	< 0.001
Crackles (%)	33 (17.3)	16 (21.9)	14 (40.0)	3 (4.6)	0 (0.0)	< 0.001
Laboratory tests						
WBC max (mean (SD))	9.63 (4.68)	6.26 (1.98)	10.38 (3.88)	13.19 (4.70)	8.94 (3.95)	< 0.001
N max (mean (SD))	52.11 (15.64)	52.81 (9.23)	56.66 (21.26)	49.54 (16.78)	49.71 (18.29)	0.152
L max (mean (SD))	44.37 (14.90)	44.52 (9.04)	36.82 (15.89)	49.17 (17.31)	41.07 (16.92)	0.001
HB max (mean (SD))	128.22 (25.62)	136.55 (35.01)	124.51 (8.61)	122.83 (19.46)	121.11 (8.77)	0.005
PLT max (mean (SD))	303.12 (113.57)	230.90 (64.88)	335.94 (85.50)	371.38 (121.21)	285.67 (116.81)	< 0.001
CRP max (mean (SD))	7.54 (11.35)	4.39 (1.99)	10.50 (12.70)	8.89 (13.17)	9.64 (19.40)	0.022
PCT (mean (SD))	0.37 (1.30)	0.10 (0.19)	0.27 (0.42)	0.58 (1.79)	0.95 (2.33)	0.034
GLB (mean (SD))	27.12 (5.98)	30.91 (6.64)	26.64 (4.63)	23.96 (3.10)	24.09 (4.83)	< 0.001
TBIL (mean (SD))	7.26 (2.90)	8.23 (3.22)	6.74 (2.50)	6.54 (2.60)	6.93 (2.36)	0.003
ALT (mean (SD))	21.09 (10.99)	23.16 (6.69)	21.06 (7.24)	19.58 (15.89)	18.17 (8.33)	0.165
AST (mean (SD))	35.72 (15.44)	31.30 (9.29)	31.51 (8.20)	41.65 (20.94)	40.39 (15.33)	< 0.001
PAB (mean (SD))	48.10 (40.33)	30.79 (15.96)	27.05 (10.59)	52.24 (17.36)	144.24 (60.73)	< 0.001
CR (mean (SD))	31.36 (12.65)	29.43 (10.00)	28.97 (12.65)	36.71 (14.97)	24.50 (3.70)	< 0.001
BUN (mean (SD))	4.14 (2.25)	5.35 (3.05)	3.49 (1.17)	3.47 (0.90)	2.87 (0.78)	< 0.001
LDH (mean (SD))	291.02 (106.98)	242.92 (66.75)	303.71 (90.62)	324.77 (127.64)	339.50 (119.01)	< 0.001
CK (mean (SD))	83.52 (63.01)	41.25 (27.13)	104.20 (70.40)	114.77 (58.73)	101.94 (75.57)	< 0.001
CKMB (mean (SD))	30.43 (16.84)	28.16 (6.14)	30.85 (25.88)	33.62 (19.23)	27.28 (14.36)	0.231
TG (mean (SD))	0.75 (0.58)	0.37 (0.37)	0.75 (0.49)	1.09 (0.61)	1.01 (0.41)	< 0.001
IGE (mean (SD))	162.48 (230.03)	NaN (NA)	78.10 (67.96)	56.95 (65.07)	211.71 (271.47)	0.387
EOS high (%)	18 (9.4)	1 (1.4)	12 (34.3)	0 (0.0)	5 (27.8)	< 0.001
Imaging						
Multilobar infiltration (%)	10 (5.2)	2 (2.7)	6 (17.1)	1 (1.5)	1 (5.6)	0.005
Pulmonary consolidation (%)	32 (16.8)	4 (5.5)	8 (22.9)	8 (12.3)	12 (66.7)	< 0.001
Atelectasis (%)	35 (18.3)	5 (6.8)	8 (22.9)	4 (6.2)	18 (100.0)	< 0.001
Pleural effusion (%)	4 (2.1)	0 (0.0)	0 (0.0)	1 (1.5)	3 (16.7)	< 0.001
Pneumomeningitis (%)	10 (5.2)	2 (2.7)	2 (5.7)	2 (3.1)	4 (22.2)	0.007
Pathogenicity						
TNGS - Bocavirus (%)	191 (100.0)	73 (100.0)	35 (100.0)	65 (100.0)	18 (100.0)	NA
TNGS - Streptococcus pneumoniae (%)	16 (8.4)	11 (15.1)	4 (11.4)	1 (1.5)	0 (0.0)	0.016
TNGS - Moraxella catarrhalis (%)	11 (5.8)	7 (9.6)	3 (8.6)	1 (1.5)	0 (0.0)	0.126
TNGS - Haemophilus influenzae (%)	11 (5.8)	3 (4.1)	7 (20.0)	1 (1.5)	0 (0.0)	0.001
Nucleic acid - Bocavirus (%)	98 (51.3)	56 (76.7)	14 (40.0)	13 (20.0)	15 (83.3)	< 0.001
Nucleic acid - Rhinovirus (%)	4 (2.1)	2 (2.7)	0 (0.0)	0 (0.0)	2 (11.1)	0.024
Nucleic acid - Parainfluenza virus (%)	2 (1.0)	1 (1.4)	1 (2.9)	0 (0.0)	0 (0.0)	0.56
Nucleic acid - Respiratory syncytial virus (%)	3 (1.6)	0 (0.0)	1 (2.9)	2 (3.1)	0 (0.0)	0.427
Single detection (%)	151 (79.1)	56 (76.7)	22 (62.9)	55 (84.6)	18 (100.0)	0.008
Mixed bacterial detection (%)	35 (18.3)	17 (23.3)	13 (37.1)	5 (7.7)	0 (0.0)	< 0.001
Mixed viral detection (%)	7 (3.7)	0 (0.0)	1 (2.9)	6 (9.2)	0 (0.0)	0.026
Mixed bacterial and viral detection (%)	2 (1.0)	0 (0.0)	1 (2.9)	1 (1.5)	0 (0.0)	0.528
Treatment						< 0.001
Penicillin class (%)	35 (18.3)	1 (1.4)	9 (25.7)	19 (29.2)	6 (33.3)	< 0.001
Cephalosporin class (%)	83 (43.5)	36 (49.3)	10 (28.6)	31 (47.7)	6 (33.3)	0.145
Azithromycin (%)	90 (47.1)	52 (71.2)	12 (34.3)	18 (27.7)	8 (44.4)	< 0.001
Methylprednisolone (%)	94 (49.2)	43 (58.9)	18 (51.4)	23 (35.4)	10 (55.6)	0.044
Symptomatic treatment (%)	9 (4.7)	1 (1.4)	6 (17.1)	0 (0.0)	2 (11.1)	

### Comparison of tNGS and nucleic acid testing

**Fig. 1:**
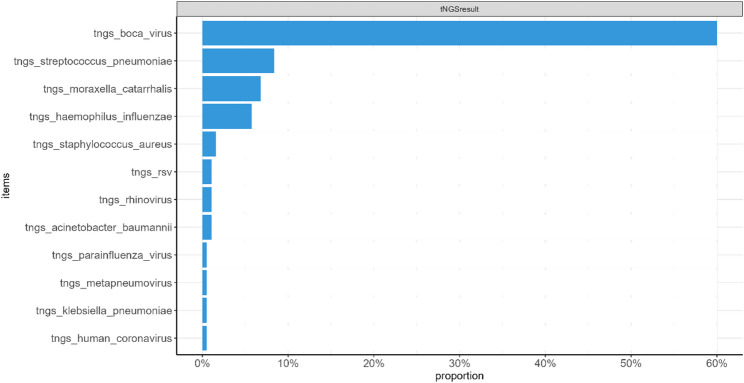
Pathogens detected by tNGS

All patients tested positive for HBoV by tNGS (100.0%), whereas nucleic acid testing detected HBoV in 51.3% (98/191) of cases (*P* < 0.001). tNGS identified 44 cases of co-detection (23.0%), including 35 bacterial co-detections (18.3%)—primarily Streptococcus pneumoniae (16, 8.4%), Moraxella catarrhalis (11, 5.8%), Haemophilus influenzae (11, 5.8%), Staphylococcus aureus (3, 1.6%), Acinetobacter baumannii (2, 1.0%), and Klebsiella pneumoniae (1, 0.5%)—and 7 viral co-detections (3.7%), including RSV (2, 1.0%), rhinovirus (1, 0.5%), parainfluenza virus (1, 0.5%), metapneumovirus (1, 0.5%), and human coronavirus (1, 0.5%) (Fig. 1).

**Fig. 2:**
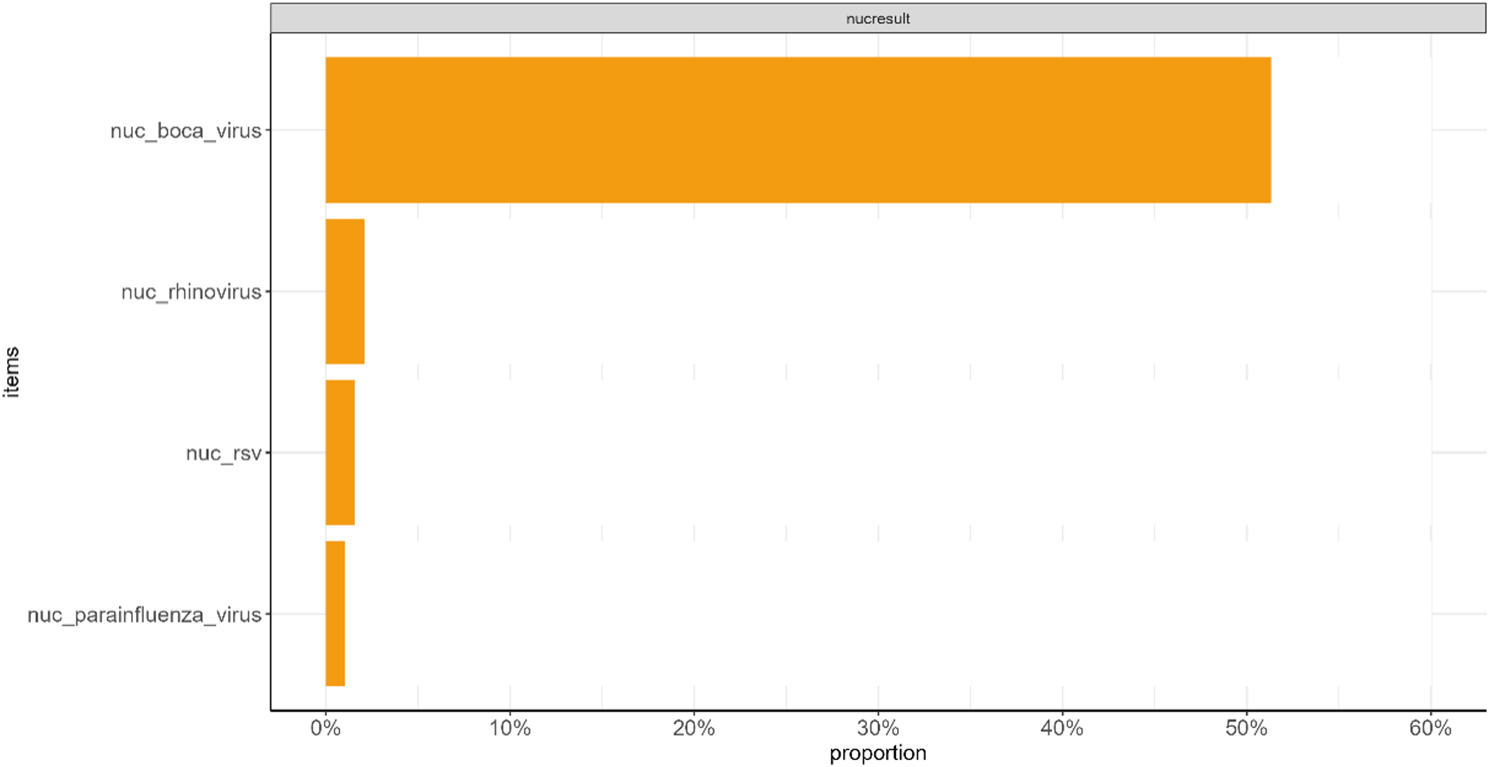
Pathogens detected by conventional nucleic acid testing

In contrast, nucleic acid testing detected only 9 viral co-detections (4.7%), including rhinovirus (4, 2.1%), RSV (3, 1.6%), and parainfluenza virus (2, 1.0%) (Fig. 2).

**Fig. 3:**
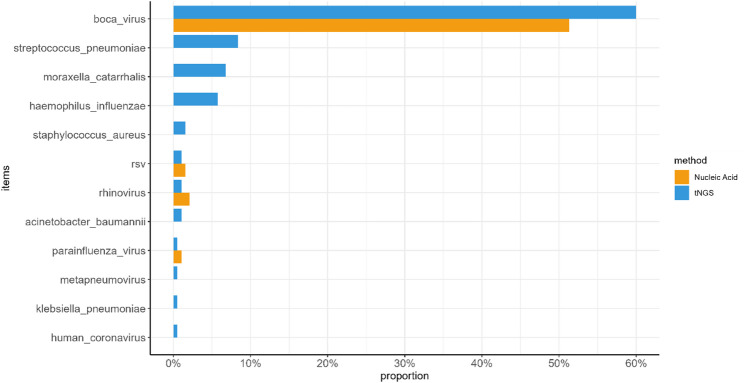
Comparison of pathogen detection rates between tNGS and nucleic acid testing

Overall, tNGS demonstrated a significantly higher detection rate for co-detections than nucleic acid testing (*P* < 0.001), particularly for bacterial pathogens. Nucleic acid testing showed slightly higher detection rates for rhinovirus, RSV, and parainfluenza virus compared with tNGS (Fig. 3).

### Comparison of tNGS and nucleic acid testing across centers

HBoV was detected by tNGS in all patients across the four centers, whereas nucleic acid testing positivity varied significantly (*P* < 0.001): highest at ZCH (83.3%, 15/18), followed by DY (76.7%, 56/73), JH (40.0%, 14/35), and lowest at YK (20.0%, 13/65).

Patterns of co-detection and pathogen detection differed among centers. At DY and JH, tNGS primarily identified S. pneumoniae, M. catarrhalis, and H. influenzae, with no viral co-detections detected; nucleic acid testing detected parainfluenza virus, rhinovirus, and RSV. At YK, tNGS detected multiple bacterial and viral pathogens, including S. pneumoniae, M. catarrhalis, H. influenzae, RSV, and rhinovirus, whereas nucleic acid testing detected only two RSV cases (3.1%). At ZCH, tNGS detected HBoV exclusively, while nucleic acid testing identified two rhinovirus cases (11.1%) (Supplementary Materials S2 and S3).

### Comparison of clinical characteristics between SCAP and non-SCAP patients

Among the 191 patients, 42 (22.0%) developed SCAP. There were no significant differences in sex, mean age, or BMI between SCAP and non-SCAP groups (*P* > 0.05). However, SCAP cases were predominantly aged 1–3 years (76.2%), whereas non-SCAP patients were mainly 1–3 years (53.7%) and 3–6 years (30.2%). SCAP most frequently occurred in summer (61.9%). Baseline clinical characteristics are summarized in Table 2.

While fever and cough were comparable between groups (*P* > 0.05), SCAP patients had higher rates of wheezing (54.8% vs. 24.8%) and dyspnea (45.2% vs. 9.4%) compared with non-SCAP patients (*P* < 0.001). Inflammatory markers, including CRP, PCT, and WBC, did not differ significantly (*P* > 0.05).

Radiologically, SCAP patients showed higher incidences of pulmonary consolidation (35.7% vs. 11.4%), atelectasis (40.5% vs. 12.1%), and pleural effusion (7.1% vs. 0.7%), as well as higher rates of plastic bronchitis on bronchoscopy (21.4% vs. 0.7%) (all *P* < 0.05). Corticosteroid use was more frequent in SCAP patients (73.8% vs. 42.3%; *P* < 0.05), while the use of other antibiotics was comparable (*P* > 0.05).

**Table 2:** Clinical characteristics of children with SCAP and non-SCAP

	Overall	non-SCAP	SCAP	*P*
n	191	149	42	
Center (%)				< 0.001
DY	73 (38.2)	65 (43.6)	8 (19.0)	
JH	35 (18.3)	22 (14.8)	13 (31.0)	
YK	65 (34.0)	53 (35.6)	12 (28.6)	
ZCH	18 (9.4)	9 (6.0)	9 (21.4)	
Age (months) (mean (SD))	25.63 (20.21)	26.12 (20.92)	23.91 (17.57)	0.534
Age Group, years (n (%))				0.036
< 1	23 (12.0)	20 (13.4)	3 (7.1)	
1–3	112 (58.6)	80 (53.7)	32 (76.2)	
3–6	50 (26.2)	45 (30.2)	5 (11.9)	
> 6	6 (3.1)	4 (2.7)	2 (4.8)	
Sex (Girl) (%)	66 (34.6)	54 (36.2)	12 (28.6)	0.46
BMI ((SD))	16.73 (2.50)	16.86 (2.60)	16.26 (2.06)	0.164
Morbidity season (%)				0.057
Spring	34 (17.8)	25 (16.8)	9 (21.4)	
Summer	92 (48.2)	66 (44.3)	26 (61.9)	
Autumn	49 (25.7)	43 (28.9)	6 (14.3)	
Winter	16 (8.4)	15 (10.1)	1 (2.4)	
Allergy history (%)	10 (5.2)	8 (5.4)	2 (4.8)	1
Oxygen inhalation (%)	35 (18.3)	11 (7.4)	24 (57.1)	< 0.001
clinical picture				
Fever (%)	159 (83.2)	124 (83.2)	35 (83.3)	1
Number of fever days (MEAN (SD))	2.49 (2.63)	2.43 (2.38)	2.70 (3.39)	0.559
Highest body temperature (MEAN (SD))	38.53 (1.05)	38.49 (1.04)	38.68 (1.09)	0.295
Cough (%)	188 (98.4)	146 (98.0)	42 (100.0)	0.822
Coughing with wheezing (%)	60 (31.4)	37 (24.8)	23 (54.8)	< 0.001
Difficulty in breathing (%)	33 (17.3)	14 (9.4)	19 (45.2)	< 0.001
Wheezing sound (%)	78 (40.8)	56 (37.6)	22 (52.4)	0.122
Rales (wet rales) (%)	86 (45.0)	62 (41.6)	24 (57.1)	0.107
Crackles (tympanic rales) (%)	33 (17.3)	29 (19.5)	4 (9.5)	0.203
Laboratory tests				
WBC_max (mean (SD))	9.63 (4.68)	9.35 (4.80)	10.59 (4.12)	0.131
N_max (mean (SD))	52.11 (15.64)	50.53 (14.72)	57.73 (17.62)	0.008
L_max (mean (SD))	44.37 (14.90)	45.33 (13.97)	40.96 (17.60)	0.094
HB_max (mean (SD))	128.22 (25.62)	129.93 (28.19)	122.14 (11.11)	0.082
PLT_max (mean (SD))	303.12 (113.57)	291.36 (106.29)	344.86 (129.24)	0.007
CRP_max (mean (SD))	7.54 (11.35)	6.95 (10.25)	9.62 (14.58)	0.178
PCT (mean (SD))	0.37 (1.30)	0.36 (1.24)	0.40 (1.52)	0.878
GLB (mean (SD))	27.12 (5.98)	27.60 (6.00)	25.40 (5.63)	0.035
TBIL (mean (SD))	7.26 (2.90)	7.16 (2.90)	7.60 (2.91)	0.385
ALT (mean (SD))	21.09 (10.99)	21.02 (11.88)	21.33 (7.13)	0.871
AST (mean (SD))	35.72 (15.44)	35.66 (16.38)	35.90 (11.64)	0.929
PAB (mean (SD))	48.10 (40.33)	44.19 (36.85)	61.95 (48.82)	0.011
CR (mean (SD))	31.36 (12.65)	30.44 (12.25)	34.62 (13.64)	0.059
BUN (mean (SD))	4.14 (2.25)	4.22 (2.21)	3.84 (2.36)	0.329
LDH (mean (SD))	291.02 (106.98)	285.96 (110.51)	308.95 (92.33)	0.219
CK (mean (SD))	83.52 (63.01)	80.97 (63.46)	92.57 (61.28)	0.293
CKMB (mean (SD))	30.43 (16.84)	30.95 (18.48)	28.56 (8.80)	0.418
TG (mean (SD))	0.75 (0.58)	0.74 (0.60)	0.78 (0.50)	0.704
IGE (mean (SD))	162.48 (230.03)	203.84 (299.30)	108.70 (64.91)	0.337
EOS high (%)	18 (9.4)	13 (8.7)	5 (11.9)	0.746
Imaging findings				
Multilobar infiltration (%)	10 (5.2)	5 (3.4)	5 (11.9)	0.071
Pulmonary consolidation (%)	32 (16.8)	17 (11.4)	15 (35.7)	< 0.001
Atelectasis (%)	35 (18.3)	18 (12.1)	17 (40.5)	< 0.001
Pleural effusion (%)	4 (2.1)	1 (0.7)	3 (7.1)	0.048
Pneumomediastinum (%)	10 (5.2)	1 (0.7)	9 (21.4)	< 0.001
Plastic bronchitis (%)				
Pathological results	191 (100.0)	149 (100.0)	42 (100.0)	NA
TNGS - Bocavirus (%)	16 (8.4)	14 (9.4)	2 (4.8)	0.521
TNGS - Streptococcus pneumoniae (%)	11 (5.8)	9 (6.0)	2 (4.8)	1
TNGS - Moraxella catarrhalis (%)	11 (5.8)	8 (5.4)	3 (7.1)	0.951
TNGS - Haemophilus influenzae (%)	98 (51.3)	74 (49.7)	24 (57.1)	0.495
Nucleic acid - Bocavirus (%)	4 (2.1)	3 (2.0)	1 (2.4)	1
Nucleic acid - Rhinovirus (%)	2 (1.0)	2 (1.3)	0 (0.0)	1
Nucleic acid - Parainfluenza virus (%)	3 (1.6)	2 (1.3)	1 (2.4)	1
Nucleic acid - Respiratory syncytial virus (%)	151 (79.1)	120 (80.5)	31 (73.8)	0.464
Single detection (%)	35 (18.3)	27 (18.1)	8 (19.0)	1
Mixed bacterial detection (%)	7 (3.7)	3 (2.0)	4 (9.5)	0.068
Mixed viral detection (%)	2 (1.0)	1 (0.7)	1 (2.4)	0.918
Mixed bacterial and viral detection (%)				
Treatment	35 (18.3)	26 (17.4)	9 (21.4)	0.717
Cephalosporins (%)	83 (43.5)	62 (41.6)	21 (50.0)	0.428
Azithromycin (%)	90 (47.1)	71 (47.7)	19 (45.2)	0.919
Methylprednisolone (%)	94 (49.2)	63 (42.3)	31 (73.8)	0.001
Symptomatic treatment (%)	9 (4.7)	6 (4.0)	3 (7.1)	0.668

**Fig. 4:**
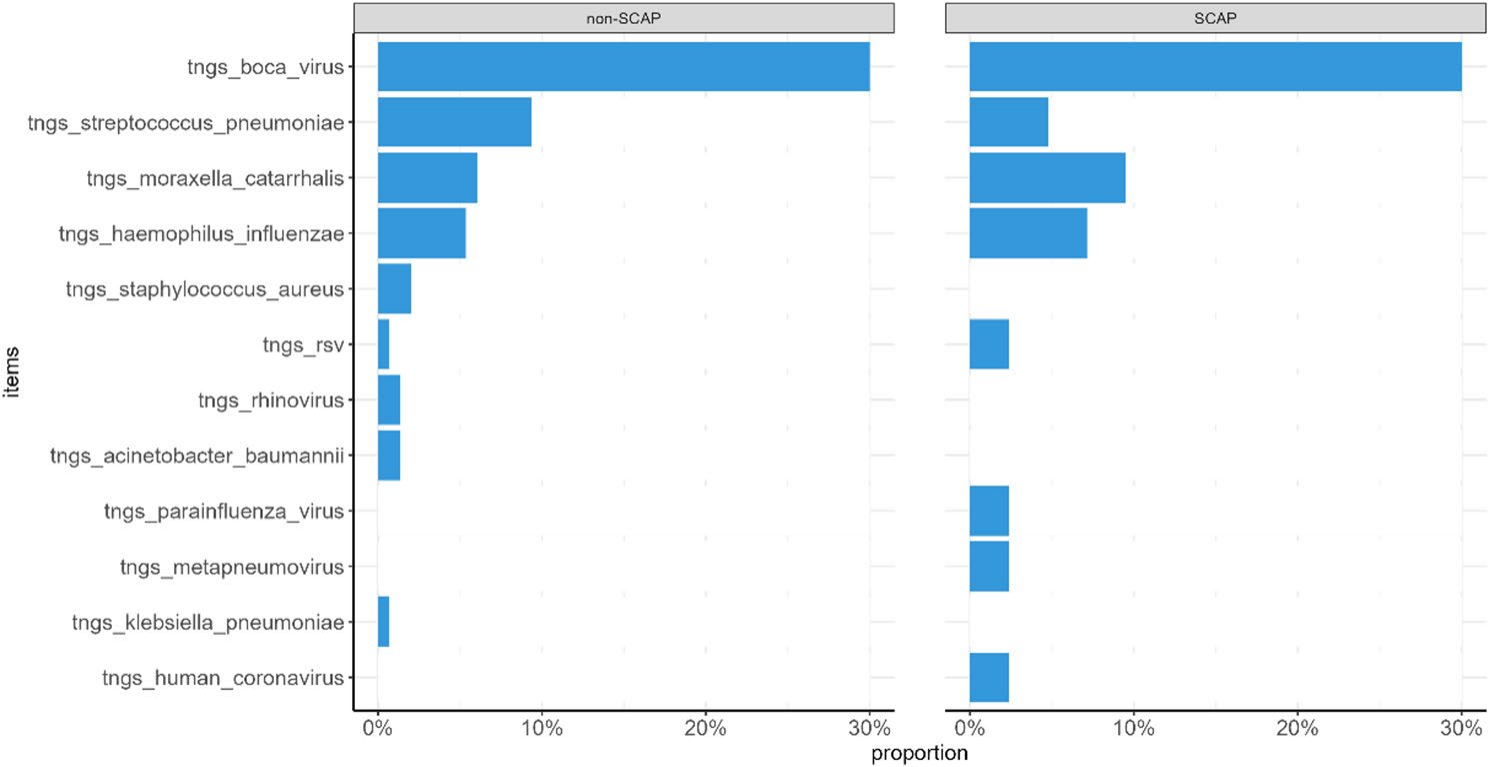
tNGS pathogen detection results in children with SCAP and non-SCAP

**Fig. 5:**
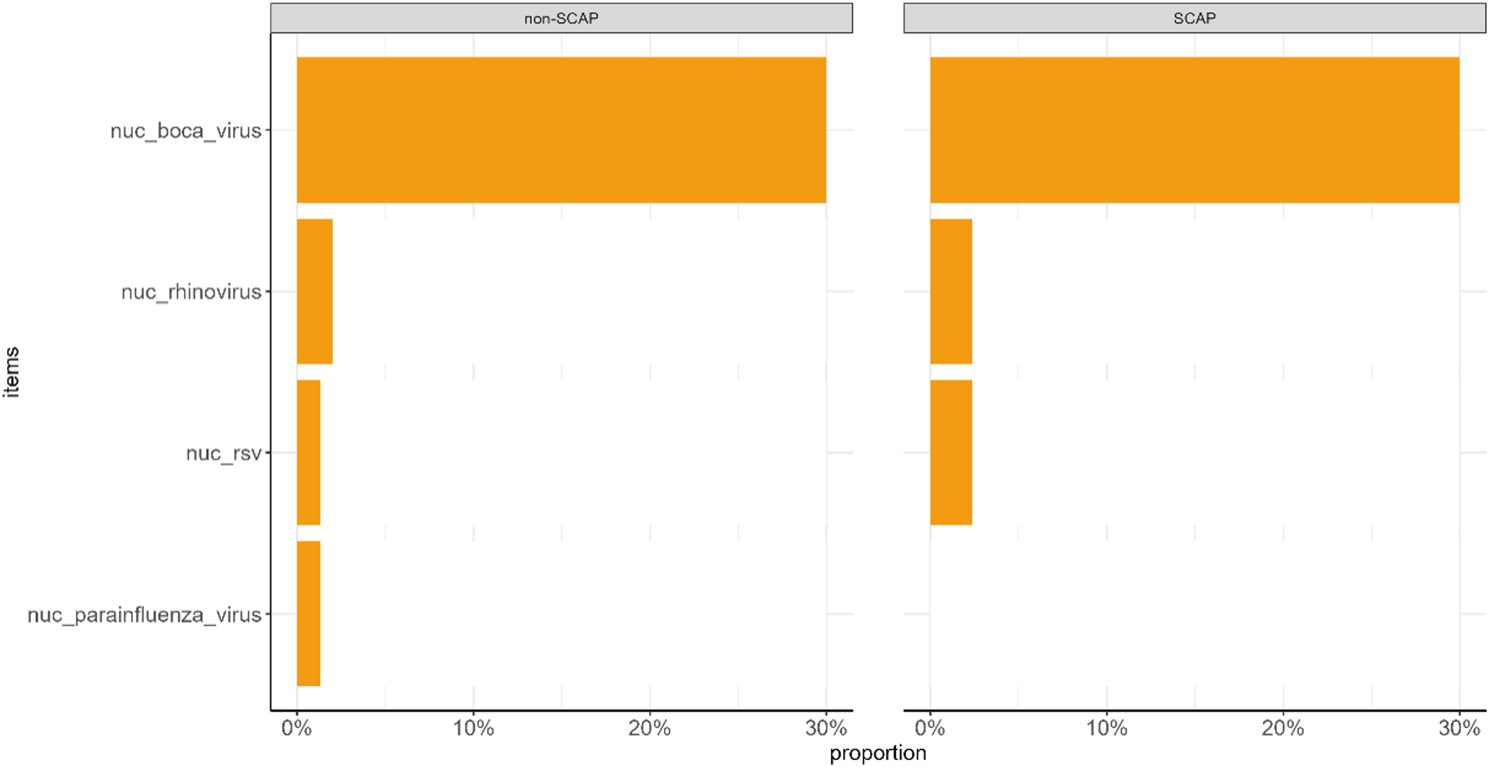
Nucleic acid testing results in children with SCAP and non-SCAP

The proportion of viral co-detection in the SCAP group (9.5%) was higher than in the non-SCAP group (2.0%), but the difference was not statistically significant. No significant differences were observed between the two groups in rates of single detection or mixed bacterial detection (*P* > 0.05) (Figs. 4 and 5).

### Risk factors for SCAP

Univariable logistic regression identified the following variables as significantly associated with SCAP: age < 3 years, co-detection with other respiratory viruses, increased lymphocyte percentage, elevated platelet count, reduced globulin, increased prealbumin, and plastic bronchitis (*P* < 0.05).

After adjusting for confounders, multivariable logistic regression showed that age < 3 years (OR = 3.036, 95% CI: 1.073–9.840, *P* = 0.047), plastic bronchitis (OR = 47.675, 95% CI: 5.883-1284.754, *P* = 0.002), and increased prealbumin (OR = 1.002, 95% CI: 0.985–1.020, *P* = 0.796) were independent risk factors for SCAP (Table 3).

**Table 3:** Risk factors of SCAP

	Model 1			Model 2			Model 3		
Predictors	Odds Ratios	CI	P	Odds Ratios	CI	P	Odds Ratios	CI	P
< 3 years old	2.450 *	1.068–6.368	**0.046**	3.115 *	1.218–9.132	**0.025**	3.036 *	1.073–9.840	**0.047**
Mixed with other viral detections	5.123 *	1.086–26.937	**0.037**	4.336	0.803–25.735	0.087	4.451	0.789–28.064	0.092
Lymphocyte ratio	1.032 **	1.008–1.058	**0.009**	1.026	1.000–1.055	0.055	0.958	0.875–1.042	0.336
Platelet count	1.004 **	1.001–1.007	**0.008**	1.003	0.999–1.008	0.096	1.003	0.999–1.008	0.174
Globulin	0.928 *	0.863–0.991	**0.035**	0.925	0.850–0.999	0.057	0.958	0.875–1.042	0.336
Prealbumin	1.009 *	1.002–1.017	**0.016**	1.014 **	1.004–1.024	**0.005**	1.002	0.985–1.020	0.796
Plastic bronchitis	40.364 ***	7.228–757.287	**0.001**	57.077 ***	8.662–1376.242	**0.001**	47.675 **	5.883–1284.754	**0.002**

Model 1: Unadjusted

Model 2: Adjusted gender, body mass index, allergy history, white blood cell count, C-reactive protein, procalcitonin, total bilirubin, lactate dehydrogenase, creatinine, and other confounding factors

Model 3: Adjusted gender, body mass index, allergy history, white blood cell count, C-reactive protein, procalcitonin, total bilirubin, lactate dehydrogenase, creatinine, and other confounding factors including medical centers

*P < 0.05, **P < 0.01, ***P < 0.001

## Discussion

This multicenter study analyzed 191 children with tNGS-confirmed Bocavirus-positive CAP, characterizing clinical heterogeneity, comparing pathogen detection concordance between tNGS and nucleic acid testing, identifying clinical features associated with SCAP.

Children aged 1–3 years were most affected (58.6%), with 76.2% of SCAP cases in this age group, consistent with previous studies. Immature immunity and underdeveloped airway barriers may predispose them to severe inflammation, emphasizing the need for early monitoring. Bocavirus infections peaked in summer (48.2%), coinciding with high SCAP incidence (61.9%), likely related to environmental factors and increased viral transmission. Recent studies demonstrated that there are asymptomatic HBoV carriage in 15% of healthy children [10], and HBoV may be a coincidental colonizer rather than a pathogenic driver even in tNGS-positive CAP cases [11]. This aligns with our finding that 79.1% of patients had HBoV mono-detection, but we lacked data to rule out asymptomatic carriage.

Significant heterogeneity was observed across centers, likely due to differences in case mix, referral patterns, and care levels. The ZCH center had the highest SCAP rate (50.0%) and more severe complications, suggesting a higher proportion of critically ill referrals. DY and YK centers had lower SCAP incidence (11.0% and 18.5%) and higher recovery rates, reflecting earlier recognition and intervention. This heterogeneity was adjusted for in the multivariate model, but it may still limit the generalizability of results, highlighting the need for stratified analysis in future studies.

Among bocavirus-positive patients detected by tNGS, the bocavirus positive rate by nucleic acid testing was 51.3%. Due to our study design restricting enrollment to tNGS-positive cases, it is difficult to rigorously demonstrate that tNGS testing is significantly more sensitive than nucleic acid testing. Additionally, the lack of viral load, mRNA, or serological data further limits our ability to compare diagnostic performance and distinguish active bocavirus infection from persistent shedding or asymptomatic carriage.

Meanwhile, our data show that tNGS has a broader range of pathogen co-detection, particularly for bacterial pathogens—consistent with its inherent advantage of simultaneously detecting multiple pathogens without relying on predefined targets [6, 11]. However, this high detection capability also increases the likelihood of identifying commensal bacteria in the upper respiratory tract. Importantly, bacterial co-detection in nasopharyngeal swabs does not equate to active infection, as some bacteria (e.g., Streptococcus pneumoniae, Haemophilus influenzae) may be commensal colonizers of the upper respiratory tract [12]. Thus, our study’s findings on pathogen co-detection only reflect the presence of pathogens, not their pathogenicity. Future studies should integrate blood cultures, inflammatory markers, and clinical course to confirm true bacterial infection and clarify their contribution to disease severity.

In clinical practice, nucleic acid testing, with lower cost and faster turnaround, remains suitable for preliminary screening of mild cases, while tNGS is recommended for severe or diagnostically unresolved cases to guide targeted therapy.

Multivariable analysis identified age < 3 years, bronchoscopic plastic bronchitis, and increased prealbumin as independent risk factors for SCAP. Children aged < 3 years are particularly vulnerable to severe disease, likely due to immature immune function and underdeveloped airway barriers. This aligns with our observation that 76.2% of SCAP cases fell into this age group, emphasizing the need for close clinical monitoring to detect early deterioration. Plastic bronchitis strongly predicts severe disease due to airway obstruction, as supported by previous clinical analyses [13]. Early bronchoscopic intervention is recommended for suspected plastic bronchitis. While prealbumin is typically viewed as a nutritional marker, recent evidence suggests it may reflect acute-phase response or inflammation-related metabolic changes in severe infections. In our cohort, higher prealbumin levels were initially associated with SCAP in unadjusted analyses, however, after adjusting for confounding factors including study center, prealbumin no longer showed a statistically significant association with SCAP. This finding implies that prealbumin levels may be linked to center-specific factors (e.g., variations in patient referral patterns) rather than serving as a universal, center-independent risk factor for SCAP. Further research is still needed to clarify its exact mechanistic role, if any, in the pathogenesis of SCAP. Notably, although viral co-detection was not identified as an independent risk factor of SCAP after adjusting confounders, it is reported that coinfection with other viruses, a common feature of bocavirus infection [12], may further exacerbate disease severity.

These findings have important clinical implications: (1) early identification of high-risk patients (< 3 years, potential plastic bronchitis, viral co-detection); (2) optimized pathogen detection using tNGS for severe or complex cases; (3) targeted therapy, including antimicrobials, supportive care, and bronchoscopic intervention; (4) multicenter collaboration to standardize care and improve referral pathways for critically ill children.

Limitations of this study include: (1) The retrospective design and inherent selection bias due to the enrollment of only bocavirus-positive CAP children detected by tNGS limit the generalizability of the diagnostic comparison results between tNGS and conventional nucleic acid testing; (2) The lack of data on viral load, mRNA expression, and serological markers precludes the distinction between acute HBoV infection and persistent viral carriage; (3) The relatively modest sample size may compromise the statistical stability of the regression models; (4) The absence of long-term follow-up prevents the assessment of long-term outcomes in children with bocavirus-associated CAP. Future large-scale, prospective multicenter studies are needed to validate these results and explore additional risk factors to guide clinical management.

## Conclusion

Bocavirus-associated CAP in children exhibits notable clinical heterogeneity across centers. tNGS offers a broader range of pathogen co-detection compared with conventional nucleic acid testing, supporting its use in severe or complex cases. Early recognition of high-risk patients (including children < 3 years, with potential plastic bronchitis, and viral co-detection), tailored pathogen detection, and timely targeted interventions are essential to improve outcomes. These findings provide important evidence to guide clinical management and highlight the value of multicenter collaboration in optimizing care for children with severe Bocavirus-associated CAP.

## Supplementary Information

Below is the link to the electronic supplementary material.

Supplementary Materials

## Data Availability

The datasets generated and/or analyzed during the current study are not publicly available due to patient privacy and ethical restrictions but are available from the corresponding author on reasonable request.

## Abbreviations

ALT: Alanine Aminotransferase
AST: Aspartate Aminotransferase
CAP: Community-Acquired Pneumonia
CI: Confidence Interval
CRP: C-Reactive Protein
CT: Computed Tomography
DY: Dongyang Women and Children’s Hospital
HBoV: Human Bocavirus
IgE: Immunoglobulin E
IQR: Interquartile Range
JH: Jinhua Women and Children’s Hospital
LDH: Lactate Dehydrogenase
OR: Odds Ratio
PCT: Procalcitonin
SCAP: Severe Community-Acquired Pneumonia
SD: Standard Deviation
tNGS: Targeted Next-Generation Sequencing
WBC: White Blood Cell Count
YK: Yongkang Women and Children’s Hospital
ZCH: Zhejiang University School of Medicine Children’s Hospital
