# Supplementary material for: Etiological characteristics and risk factors for severe disease in bocavirus-associated community-acquired pneumonia in children: a multicenter retrospective study
Source: BMC Infect Dis. 2026 Jan 27;26:400. doi: 10.1186/s12879-026-12543-z (PMC12918134; doi:10.1186/s12879-026-12543-z)
Supplement: Supplementary file 1 — Supplementary Materials [file 12879_2026_12543_MOESM1_ESM.docx]

**Supplementary Materials**

**Supplementary Table S1 Characteristics of the participating medical centers.**

| **Center**  **(Abbreviation)** | **Level of Care & Hospital Type** | **Catchment Area / Service Population** | **PICU & Specialized Services** | **Typical Patient Population / Referral Pattern** |
| --- | --- | --- | --- | --- |
| Children’s Hospital, Zhejiang University School of Medicine (ZCH) | National-level tertiary grade-A hospital/regional referral center. | Accept referrals from across the region and the rest of the country. | Yes./ Fully equipped, multidisciplinary PICU, EICU, NICU, CICU, SICU; advanced respiratory support (e.g., HFOV, ECMO consultation). | Predominantly severe, complex, or treatment-resistant cases transferred from lower-tier hospitals. |
| Yongkang Maternal and Child Health Hospital (YK) | National-level tertiary Grade B hospital / regional maternal and child health hospital. | Serves Yongkang city and its districts. | Yes./PICU and the Critical Neonatal Consultation and Treatment Center | Mainly community-acquired cases from the local area; spectrum includes mild, moderate, and initial presentation of severe cases. |
| Jinhua Maternal and Child Health Hospital(JH) | National-level tertiary grade-A hospital / regional maternal and child health hospital. | Serves Jinhua city and its surrounding counties. | Yes./Comprehensive ICU, NICU; Advanced respiratory support (such as ECMO). | Primarily community-acquired cases from the local area; spectrum includes mild, moderate, and initial severe cases. |
| Dongyang Maternal and Child Health Hospital(DY) | National-level tertiary Grade B hospital / regional maternal and child health hospital. | Serves Dongang city and its surrounding counties. | Yes./Comprehensive ICU, NICU; Advanced respiratory support (such as HFOV,). | Primarily community-acquired cases from the local area; spectrum includes mild, moderate, and initial severe cases. |


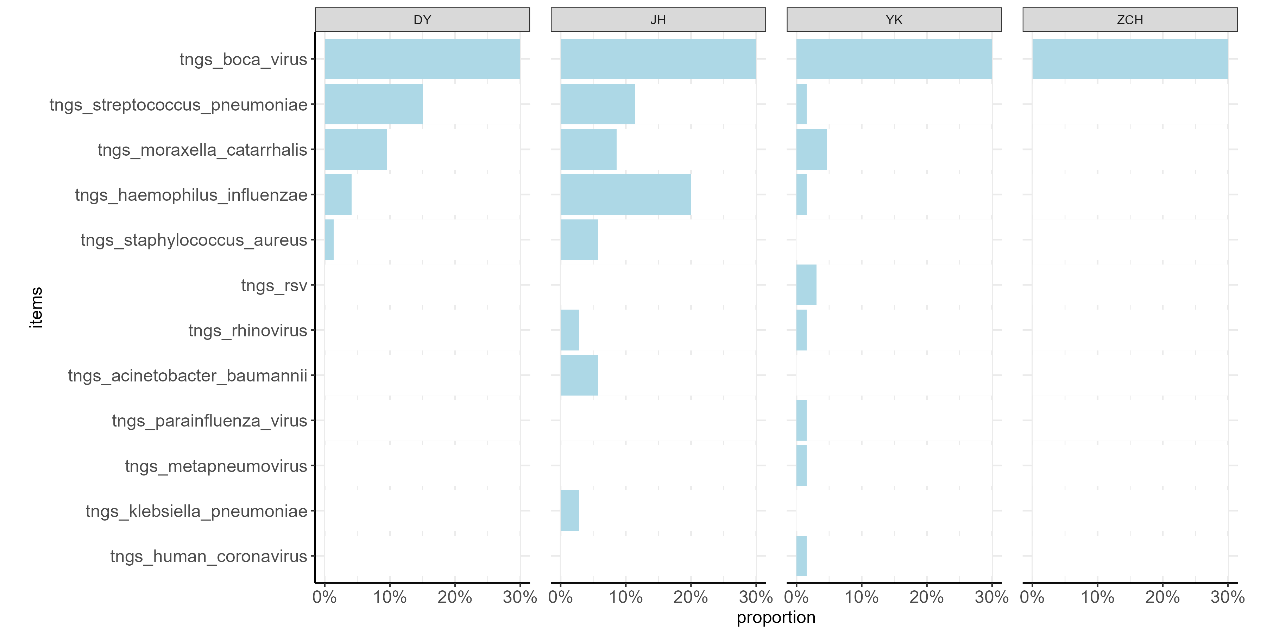
**Supplementary Materials S2** Pathogens detected by tNGS across four centers


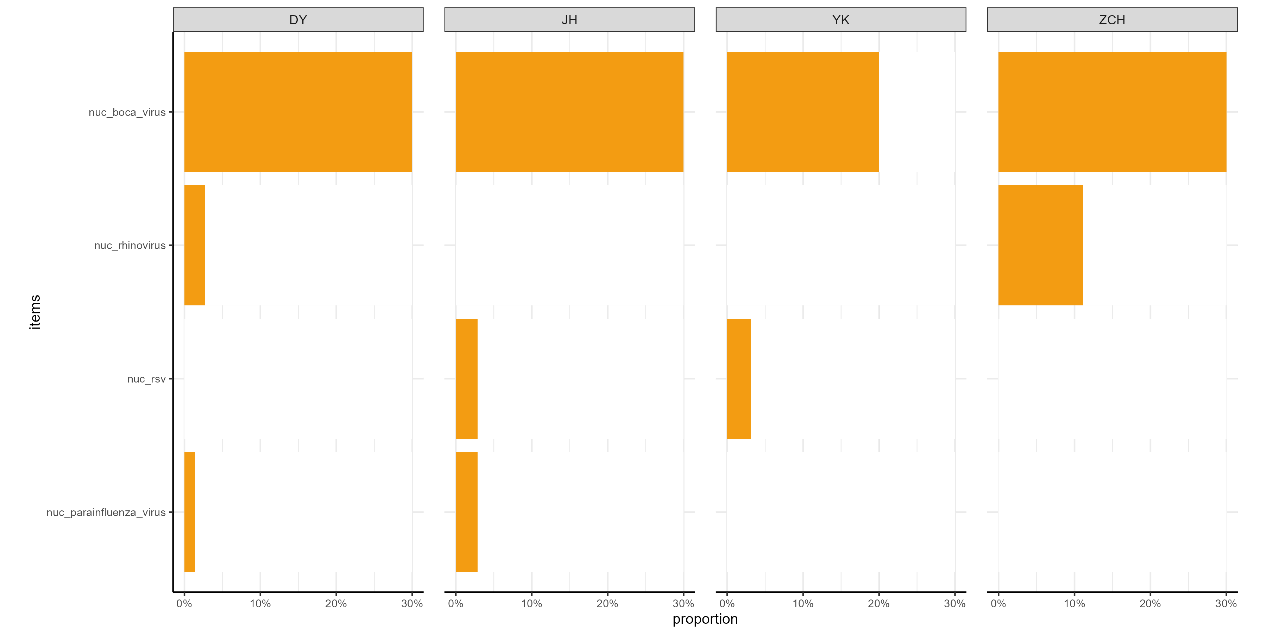


**Supplementary Materials S3** Pathogens detected by conventional nucleic acid testing across four centers
